# PML as a neuroprotective guardian: Leveraging nuclear protein quality control to mitigate neurotoxicity of an ALS‐associated NEK1 variant

**DOI:** 10.1111/febs.70630

**Published:** 2026-06-19

**Authors:** Tabea Stark, Stefan Müller

**Affiliations:** ^1^ Institute of Biochemistry II Goethe University Frankfurt, Faculty of Medicine Frankfurt Germany; ^2^ Cluster of Excellence SubCellular Architecture of Life (SCALE), Goethe University Frankfurt Frankfurt Germany

**Keywords:** ALS, NEK1, PML, SUMO, SUMO‐targeted ubiquitylation, ubiquitin

## Abstract

Insoluble protein aggregates are a hallmark of neurodegenerative diseases like amyotrophic lateral sclerosis (ALS). The ubiquitin–proteasome system (UPS) serves as a neuroprotective quality control mechanism that clears aggregates. PML nuclear bodies (NBs) were proposed to serve as hubs for SUMO‐primed ubiquitylation and degradation of misfolded proteins. Georgiadou *et al.* provide evidence that an ALS‐linked NEK1 truncation mutant is recruited to PML NBs, where it likely undergoes SUMOylation and ubiquitylation. In mice, PML loss exacerbates ALS‐like symptoms, while induced PML expression delays disease onset. These findings establish PML as a key regulator of proteostasis and highlight PML induction as a potential therapeutic strategy for ALS and related proteinopathies.

## Graphical abstract

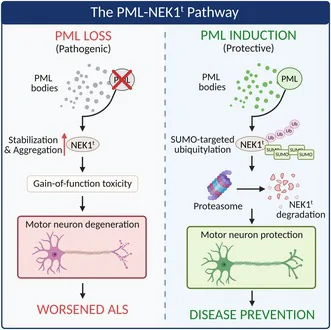

**Abbreviations**
ALS: amyotrophic lateral sclerosis
PML NBs: Promyleocytic leukemia nuclear bodies
StUbLs: SUMO‐targeted ubiquitin ligases
SUMO: small ubiquitin‐related modifier
UPS: ubiquitin proteasome system

## Introduction

Protein misfolding induced by proteotoxic stress or genetic mutations poses a significant threat to cellular function and organismal health [1]. Misfolding of critical regulatory proteins can lead to either loss‐ or gain‐of‐function toxicity. Gain‐of‐function toxicity of misfolded proteins is frequently associated with the formation of insoluble protein aggregates, a hallmark of a broad class of diseases known as proteinopathies, including neurodegenerative disorders such as polyglutamine (polyQ) diseases, Alzheimer's disease, and amyotrophic lateral sclerosis (ALS) [2]. ALS is a fatal neurodegenerative condition characterized by the progressive degeneration of upper and lower motor neurons, leading to muscle atrophy, paralysis, and ultimately respiratory failure. While the majority of ALS cases are sporadic, approximately 10% are familial, driven by mutations in genes such as *SOD1*, *C9ORF72*, *TARDBP* (encoding TDP‐43), and *FUS*. A common pathophysiological thread among these genetic variants is the disruption of protein homeostasis (proteostasis), particularly by promoting protein misfolding and aggregation. Notably, mutations in *TARDBP* and *FUS* enhance the cytoplasmic mislocalization and aggregation of TDP‐43 and FUS, respectively—processes central to ALS pathology and associated with the formation of stress granules and pathological inclusions.

To counteract proteotoxic stress and prevent the accumulation of misfolded proteins, cells activate a comprehensive network of protein quality control (PQC) systems. Molecular chaperones assist in refolding misfolded proteins, while degradation pathways—primarily the ubiquitin–proteasome system (UPS) and the autophagy–lysosome pathway—eliminate irreversibly damaged or aggregated proteins [2]. The UPS is a central pillar of PQC, with ubiquitylation serving as the primary signal for proteasomal degradation. E3 ubiquitin ligases are the key determinants of substrate specificity, ensuring that appropriate targets are marked for destruction. A specialized subset of E3 ligases, known as SUMO‐targeted ubiquitin ligases (StUbLs)—including RNF4, RNF111, and TOPORS—plays a critical role in the selective degradation of SUMOylated proteins [3]. These ligases recognize proteins modified by SUMO (Small Ubiquitin‐like Modifier) and catalyze their ubiquitylation, thereby linking SUMOylation to proteasomal degradation. This “SUMO‐ubiquitin crosstalk” is particularly important in the nucleus, where it helps maintain proteostasis by clearing damaged or misfolded proteins [4, 5, 6].

Emerging evidence indicates that PML nuclear bodies (PML NBs) serve as functional hubs for StUbL‐mediated degradation and PQC [7]. These dynamic, phase‐separated nuclear compartments, which are defined by their scaffolding protein PML, concentrate SUMOylated substrates, StUbLs, and components of the ubiquitin–proteasome machinery. The current model posits that misfolded or damaged nuclear proteins that become SUMOylated are recruited to PML NBs, where they undergo SUMO‐primed ubiquitylation and subsequent proteasomal degradation.

### An ALS‐associated NEK1 truncation is sequestered in SUMO‐positive nuclear structures

Exome sequencing and genome‐wide association studies have identified NEK1 (NIMA‐related kinase 1) as an ALS‐associated gene in both familial and sporadic ALS [8, 9, 10]. NEK1 is a serine/threonine kinase involved in critical cellular processes, including DNA damage response, cilia function, mitotic regulation, and maintenance of genomic stability. Emerging evidence indicates that NEK1 plays a particularly important role in the integrity of the cytoskeleton and axonal transport—processes that are severely disrupted in ALS. Mono‐allelic variants of NEK1, including point mutations and truncations, were found in around 3% of both familial and sporadic ALS cases. Most ALS‐associated NEK1 variants were proposed to be heterozygous loss‐of‐function mutants, arguing in favor of NEK1 haploinsufficiency as the molecular genetic mechanism. However, it has not been determined whether distinct mutants may also function as dominant negative variants that exert gain‐of‐function toxicity. In their study, Georgiadou *et al*. followed the idea that protein aggregation may contribute to the toxicity of NEK1 mutants [11]. Along this line, they focused on the characterization of an ALS‐associated NEK1 variant that harbors a premature stop codon due to an Arg812Ter mutation. The truncated NEK1 (NEK1^t^) lacks the C‐terminal portion of the protein, containing two NES sequences. Initial analysis in cultured human cells revealed that—in contrast to the cytoplasmic localization of wild‐type NEK1, NEK1^t^ exhibits a nuclear staining with a concentration in nuclear puncta, where it co‐localizes with SUMO. Consistent with this finding, NEK1^t^ is targeted by modification with SUMO. Importantly, biochemical fractionation demonstrates that, in contrast to the soluble NEK1, NEK1^t^ is largely insoluble, potentially indicating that the nuclear foci correspond to protein inclusions or aggregates. However, a more detailed biophysical characterization remains to determine whether the puncta correspond to aggregation‐prone assemblies, biomolecular condensates, or irreversible aggregates.

### PML expression controls NEK1^t^ turnover and ALS‐penetrance in a mouse model

Considering that PML NBs function as a hub for SUMOylation, the authors further explored a physical and functional link between NEK1^t^ and PML. While only a moderate co‐localization of NEK1^t^ with PML NBs is found under basal conditions, interferon (IFNα) treatment, which strongly induces the expression of PML, triggers the recruitment of NEK1^t^ to enlarged PML NBs. Furthermore, IFNα fosters the SUMOylation of NEK1^t^ along with its proteasome‐dependent degradation. Altogether, these data are in line with the concept that induction of PML leverages NB‐associated SUMOylation of NEK1^t^ followed by its StUbL‐mediated degradation. Although this order of events would be largely consistent with the emerging role of a PML‐SUMO‐StUbL axis in nuclear PQC, future studies need to formally demonstrate the involvement of StUbLs for the clearance of NEK1^t^. Furthermore, the exact biochemical role of PML in the SUMOylation process and clearance remains to be determined [12]. Despite these open mechanistic questions, Georgiadou *et al*. provide strong genetic *in vivo* data that support the crucial role of PML in protecting mice from NEK1^t^‐driven neurotoxicity. When bi‐allelically expressed in mice, the NEK1^t^ variant induces ALS symptoms and forms inclusions in α‐motor neurons (αMNs). Intriguingly, however, in a PML‐deficient background, even heterozygous NEK1^t^ animals develop ALS symptoms, possibly indicating that PML acts as a critical modifier of disease penetrance in NEK1‐associated ALS. Consistent with this hypothesis, PML induction by poly(I:C) treatment and activation of interferon signaling accelerates NEK1^t^ clearance in αMNs of mice, improves ALS‐associated symptoms, and significantly prolongs survival. This supports the emerging concept that PML activity can be leveraged to mitigate protein aggregation and neurodegenerative disease. A critical contribution of PML in the clearance of neurotoxic protein aggregates was first described in cellular and preclinical mouse models of spinocerebellar ataxias (SCAs) [13, 14]. Mutant forms of ataxin‐1 (in SCA1) and ataxin‐7 (in SCA7) that undergo misfolding and aggregation due to their expanded polyQ tracts interact with PML and are cleared by SUMO‐targeted ubiquitylation. Very similar to the findings of Georgiadou *et al*., PML dysfunction was shown to drive enhanced SCA7 aggregation, while treatment with interferon reduced aggregation and improved locomotion in a preclinical *SCA7* knock‐in mouse model [15].

## Conclusion

**Fig. 1:**
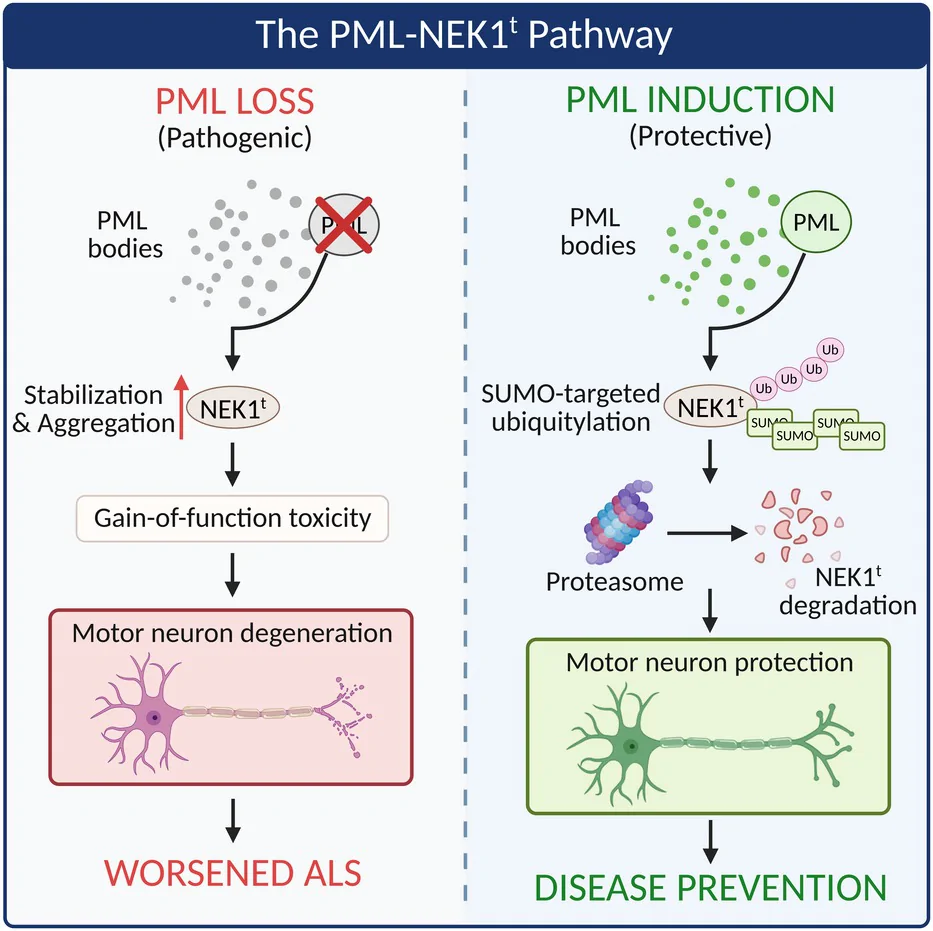
Model of the PML–NEK1^t^ quality control pathway in ALS. Left: Loss of PML disrupts PML NBs, promoting NEK1^t^ stabilization and aggregation. Toxic gain‐of‐function effects of NEK1^t^ lead to motor neuron degeneration and ALS disease progression. Right: PML induction and formation of enlarged PML NBs promote SUMO‐dependent ubiquitylation of NEK1^t^, targeting it for proteasomal degradation. This reduces NEK1^t^ toxicity, thereby preserving motor neuron integrity and delays the onset of ALS.

Altogether, the data by Georgiadou *et al*. strengthen the overall view that PML‐associated protein quality control is a disease‐relevant targetable pathway in neurodegenerative disease (Figure 1) [3, 12, 16]. Along this line, PML dysfunction has been observed in C9ORF72 and FUS‐linked cases, suggesting a broader role for PML in other familial or sporadic cases of ALS [17]. Pharmacological induction of PML expression may therefore offer therapeutic opportunities for ALS and other proteinopathies. An alternative, more selective approach to limit the formation of neurotoxic protein aggregates could be the targeted recruitment of aggregation‐prone proteins to PML [3]. Consistently, proof‐of‐concept experiments have demonstrated that proximity‐inducing modalities that tether the ALS‐associated TDP‐43 protein to PML limit the formation of TDP‐43 inclusions [16].

## Conflicts of interest

The authors declare no conflicts of interest.

## Author contributions

SM wrote the manuscript, TS edited the MS and generated the Figure.
